# Severe asphyxia due to delivery-related malpractice in Sweden 1990–2005

**DOI:** 10.1111/j.1471-0528.2007.01602.x

**Published:** 2008-02

**Authors:** S Berglund, C Grunewald, H Pettersson, S Cnattingius

**Affiliations:** a Department of Clinical Science and Education, Södersjukhuset, Karolinska Institute Stockholm, Sweden; b Department of Medical Epidemiology and Biostatistics, Karolinska Institute Stockholm, Sweden

**Keywords:** Asphyxia, cerebral palsy, delivery, fetal surveillance, hyperstimulation, malpractice, neonatal death, oxytocin, vacuum extraction

## Abstract

**Objective:**

To describe possible causes of delivery-related severe asphyxia due to malpractice.

**Design and setting:**

A nationwide descriptive study in Sweden.

**Population:**

All women asking for financial compensation because of suspected medical malpractice in connection with childbirth during 1990–2005.

**Method:**

We included infants with a gestational age of ≥33 completed gestational weeks, a planned vaginal onset of delivery, reactive cardiotocography at admission for labour and severe asphyxia-related outcomes presumably due to malpractice. As asphyxia-related outcomes, we included cases of neonatal death and infants with diagnosed encephalopathy before the age of 28 days.

**Main outcome measure:**

Severe asphyxia due to malpractice during labour.

**Results:**

A total of 472 case records were scrutinised. One hundred and seventy-seven infants were considered to suffer from severe asphyxia due to malpractice around labour. The most common events of malpractice in connection with delivery were neglecting to supervise fetal wellbeing in 173 cases (98%), neglecting signs of fetal asphyxia in 126 cases (71%), including incautious use of oxytocin in 126 cases (71%) and choosing a nonoptimal mode of delivery in 92 cases (52%).

**Conclusion:**

There is a great need and a challenge to improve cooperation and to create security barriers within our labour units. The most common cause of malpractice is that stated guidelines for fetal surveillance are not followed. Midwives and obstetricians need to improve their shared understanding of how to act in cases of imminent fetal asphyxia and how to choose a timely and optimal mode of delivery.

*Please cite this paper as:*Berglund S, Grunewald C, Pettersson H, Cnattingius S. Severe asphyxia due to delivery-related malpractice in Sweden 1990–2005. BJOG 2008;115:316–323.

## Introduction

Medical malpractice during labour is rare, but it may cause severe asphyxia, leading to death, cerebral palsy (CP) or other lifelong handicaps, with great suffering to the infants and families.^1^ The exact proportion of newborn infants who are injured during delivery is not known, but in Sweden, there are annually 20 to 50 claims for economic compensation due to suspected malpractice during pregnancy, delivery or the neonatal period. After an investigation conducted by the Patient Advisory Committee (PAC), about 50% of these cases are assessed to be a result of medical malpractice. The annual direct insurance expenditure for malpractice related to childbirth is extremely costly because of the lifelong compensation and amounts to 150 million Swedish kronor, which is equivalent to £10.7 million per annum. (K. Essinger, Managing Director at County Council Mutual Insurance Company, Sweden, pers. comm.)

In the present investigation, we describe acts of suspected malpractice in connection with childbirth as the presumable cause of severe asphyxia. For this purpose, we have scrutinised case records of births from 1990 to 2005 in Sweden filed by the PAC because of suspected malpractice with the aim of increasing our knowledge and awareness of possible causes of severe asphyxia.

## Methods

In cases of childbirth malpractice in Sweden, all affected women are entitled to compensation from the County Councils’ Mutual Insurance Company under the Patients Injury Act. The PAC (Patient Skade Reglering AB in Swedish) is responsible for the entire investigation and collects all medical documents and case records. In suspected medical injuries related to pregnancy, labour and the neonatal period, obstetricians, neonatologists, neuroradiologists and paediatric rehabilitation specialists investigate the case and assess the claims. The preliminary neurological diagnoses (i.e. ‘unspecified CP syndrome’) are based on investigations and diagnoses stated by the involved paediatricians and neurologists. With access to further clinical investigations, neuroimaging and other refined techniques, CP is later classified into subgroups, and it is also possible to time-relate the aetiology of CP in infants born at term.^2^,^3^ The classification by type of CP is made by the paediatrician or neurologist responsible for the patient. Finally, and generally, within 5 years from birth, the experts on the PAC make the final assessment concerning the diagnosis, when the damage occurred and the degree of impairment, which is based on the current Swedish disablement classification.^4^ In general, the judgement as to whether the injury was caused by malpractice is made by two obstetricians. Thereafter, the PAC decides whether or not to compensate the woman.

During the study period 1990–2005, there were about 1 622 000 live births in Sweden, on average 101 000 births per year, ranging from 86 000 to 123 000 during the period (Swedish Medical Birth Registry, http://192.137.163.40/epcfs/index.asp?modul=mfr). We reviewed all medical documentation of claims sent to the PAC from 1990 to 2005 concerning suspected malpractice during pregnancy, delivery and the neonatal period. A structured protocol was drawn up before the review, including clinical data from the standardised Swedish antenatal, obstetric and neonatal records, fetal surveillance with cardiotocographic (CTG) monitoring, fetal blood sampling (FBS), the partogram (i.e. recording of signs of dystocia during labour) and use of oxytocin. The medical review was used to find the cases with labour-related asphyxia due to malpractice. All medical documents were scrutinised and computerised by one of us (S.B.), a specialist in obstetrics during a period of 15 years and also a graduate of the American Neonatal Resuscitation Provider Program.^5^ The reviewer (S.B.) needed access to all information from each case record and therefore was not blinded to the final outcome.

Delivery-related malpractice associated with asphyxia included ‘neglecting to supervise fetal wellbeing’, ‘neglecting signs of fetal asphyxia’ and ‘malpractice around delivery’ (Table 1).

**Table 1:** The most common events of malpractice in relation to delivery causing severe asphyxia

	n (%)
**Neglecting to supervise fetal wellbeing**	173 (98)
No CTG recording after admission test	12
Uninterpretable CTG recordings (poor quality)	41
No FBS despite a clear indication	100
No follow up of previous FBS despite nonassuring CTG	20
**Neglecting signs of fetal asphyxia**	126 (71)
More than 45 minutes from onset of pathological CTG to birth	126
Increasing intravenous oxytocin infusion despite pathological CTG*	126
Hyperstimulation of uterine contractions**	61
**Malpractice around delivery**	92 (52)
Time from decision on delivery to birth exceeded 30 minutes	44
Spontaneous vaginal delivery despite long-standing (>45 minutes) pathological or uninterpretable CTG recordings	48
Traumatic instrumental delivery	44
Inadequate trial of labour	25
Too much time using the vacuum extractor to deliver (>20 minutes)***	19

* Defined according to International Federation of Obstetrics and Gynecology classifications and the Krebs intrapartum FHR scoring system.^6^

** Six or more uterine contractions/10 minutes for >20 minutes.

*** Four had more than two cup detachments with vacuum extractor.

Malpractice concerning ‘neglecting to supervise fetal wellbeing’ was defined as no CTG recording after admission test, no interpretable CTG recording after admission test because of poor quality or refraining from performing an FBS or no follow up of a previous FBS when indicated and technically possible.

With reference to malpractice due to ‘neglecting signs of fetal asphyxia’, we defined malpractice as not acting timely on pathological CTG (more than 45 minutes from onset of pathological CTG to birth), increasing oxytocin stimulation despite pathological CTG or signs of uterine hyperstimulation. A pathological CTG recording was defined according to the International Federation of Obstetrics and Gynecology classifications of CTG recordings and the Krebs intrapartum fetal heart rate (FHR) scoring system. The features that were considered pathological were a baseline FHR <100 or >170, variability amplitude of <5 beats per minutes for more than 40 minutes, severe variable-, severe repeated early-, prolonged-, late or sinusoidal decelerations.^6^–^8^ We defined hyperstimulation of uterine contractions as ≥6 contractions per 10 minutes during at least 20 minutes.^9^

‘Malpractice around delivery’ was considered to occur in cases of imminent asphyxia if the time from the decision to deliver to birth exceeded 30 minutes, if there was a spontaneous vaginal delivery despite a long-standing (at least 45 minutes) pathological or uninterpretable CTG recording or if there was a traumatic vaginal instrumental delivery. We defined a traumatic instrumental delivery as an inappropriate trial of labour with a vacuum extractor or forceps in the following circumstances: incomplete cervical dilatation, noncephalic presentation or cephalic malpresentation, nonengaged fetal head or clear indication of cephalopelvic disproportion. The definition of a traumatic instrumental delivery also included a delivery by vacuum extraction exceeding more than 20 minutes (thus considerably exceeding the recommended time of 15 minutes) or more than two cup detachments.^10^–^14^

Information about the date and time of birth, gender, birthweight, Apgar scores at 1, 5 and 10 minutes, umbilical cord pH and acts of resuscitation were retrieved from the neonatal records. We also retrieved information on the long-term follow up, including information on morbidity (CP and other neurological disorders), degree of invalidity and death from the paediatric medical records.

We included infants with a gestational age of ≥33 completed gestational weeks, a planned vaginal onset of delivery, a normal CTG tracing at admission to the delivery ward and severe asphyxia-related neurological outcomes, presumably caused by malpractice in connection with labour. We included asphyxiated–depressed infants at birth who died within 28 days and infants with severe neurological diagnoses in association with malpractice around delivery. If the acid–base status was measured in the umbilical cord at delivery or shortly thereafter, we defined asphyxia in connection with delivery as a pH from the umbilical cord at or shortly after birth of <7.05 and/or a base excess (BE) of −12 or an Apgar score of <7 at 5 minutes.^15^,^16^

**Figure 1:**
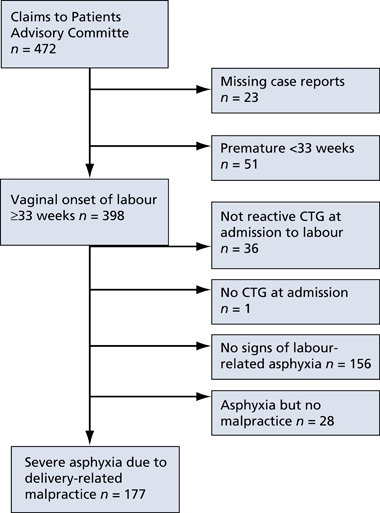
Asphyxia due to delivery-related malpractice in Sweden from 1990 to 2005.

During the study period, 472 claims pertaining to suspected delivery-related malpractice were sent to the PAC (Figure 1). Medical records were incomplete in 23 cases, and 51 infants had a gestational age of <33 completed weeks. Gestational age was estimated by an early ultrasound scan in all cases. Of the remaining 398 cases, all of which involved a vaginal onset of delivery, 36 were excluded because of pathological CTG at admission and 1 because of an absence of CTG at admission. Of the remaining 361 infants, another 156 did not have symptoms of labour-related asphyxia at birth and had a full Apgar score at 5 minutes. Twenty eight of a total of 205 cases involving labour-related asphyxia were considered to be unavoidable. This group included correctly handled complications during delivery, such as performing an immediate caesarean section shortly after admission because of a clear indication (placental abruption or a prolapsed umbilical cord) or uncomplicated deliveries with no signs of perinatal asphyxia. In all, we included 177 severe cases with labour-related asphyxia, probably due to malpractice according to our definitions. There was a high degree of concordance between our evaluation of the cases and the one made by the experts of the PAC, with a divergent assessment in 10%. These were all claims refused by the PAC, whereas we consider them to be incorrectly managed. These cases are now being reprocessed by the PAC.

All analyses were performed in SPSS 14.0 for windows 2005 (SPSS Inc., Chicago, IL, USA). The study was approved by the Research Ethics Committee at Karolinska Institute (no. 1589-2006).

## Results

All 177 mothers attended antenatal care sessions. At labour, 7% (*n* = 13) had a breech presentation and 93% (*n* = 164) a cephalic presentation. Of the 177 women who went into trial of labour, 73% (*n* = 130) had a spontaneous onset of labour and 27% (*n* = 47) were induced. The most common indications for induction were prolonged pregnancy (*n* = 14), pre-eclampsia (*n* = 9) and oligohydramniosis (*n* = 6). Twenty-seven percent (*n* = 48) were delivered spontaneously vaginally and 36% delivered by vacuum extraction (*n* = 62) or forceps (*n* = 2), while 37% (*n* = 65) delivered abdominally.

### Neglecting to supervise fetal wellbeing

All 177 cases had a reactive CTG recording at admission for labour, indicating a nonasphyxiated fetus. In 41 of 165 case records with CTG recordings after the admission test, the fetal heart tracings were not interpretable because of poor quality (Table 1). Thus, to ensure fetal wellbeing, either a change of electrodes or FBS would have been indicated. Despite a clear indication for FBS, this was not implemented in 100 cases (Table 1). There was no follow up in 20 of 37 cases with a previously performed FBS inspite of continuously pathological CTG (Table 1). Although 124 women had recordings of fetal heart tracings of adequate quality, simultaneous recording of uterine contractions was performed only in 93 cases.

In 121 of the 165 women with CTG recordings, the obstetrician was paged by the midwife because of a suspicion of pathological CTG. This occurred after more than 1 hour of pathological CTG in 50 deliveries (41%). The mean time from when the obstetrician was paged to birth was 73 minutes (range 5–464 minutes). In 11 cases, the obstetrician did not arrive in the labour room, despite having been paged numerous times. In 44 cases, the obstetrician was never paged, despite the pathological CTG.

### Neglecting signs of fetal asphyxia

**Figure 2:**
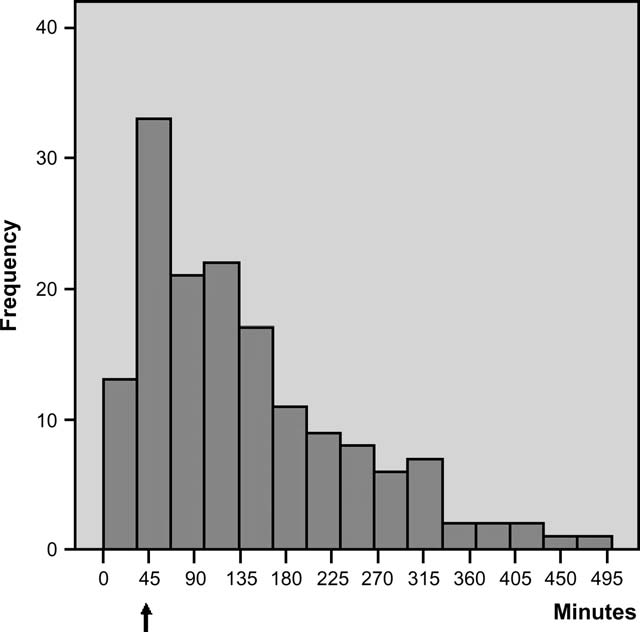
Time from onset of pathological CTG to birth (*n* = 155). Six cases are excluded in which CTG was pathological for more than 500 minutes.

In 126 deliveries, the time from the onset of pathological CTG to birth exceeded 45 minutes, indicating that the midwife and/or the obstetrician did not act timely on signs of fetal asphyxia (Table 1). The mean time from pathological CTG to birth was 160 minutes (median 118 minutes, range 9–957 minutes). Of all infants, only 16% (*n* = 29) were born within 45 minutes from the onset of pathological CTG tracings and 24% (*n* = 43) delivered within 1 hour (Figure 2).

Of the 177 women, 157 had an intravenous infusion of oxytocin lasting more than 1 hour. Of these women, 138 had an acceptable series of CTG recordings during labour, but these were of interpretable quality in only 116 cases. Simultaneous recordings of uterine contractions were available in 53% of all women receiving oxytocin infusions (*n* = 83). The dosage of intravenous oxytocin was increased for 126 women, despite pathological or noninterpretable CTG recordings (Table 1). Sixty one of the women receiving oxytocin and having pathological CTG had recordings of at least six contractions per 10 minutes registered, indicating hyperstimulation of the uterus (Table 1). For 35 women receiving oxytocin, there were indications for, but no implementation of, either a change of CTG electrodes to improve recording or FBS to ensure fetal wellbeing. Oxytocin was given without signs of uterine inertia to 49 women, 19 of whom were hyperstimulated and 44 received oxytocin, despite severely pathological heart tracings.

### Malpractice around delivery

Ninety-two women (52%) were considered to have had a nonoptimal mode of delivery (Table 1).

**Figure 3:**
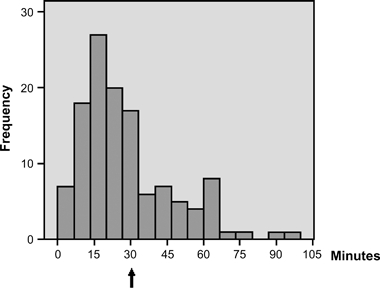
Noted time from the decision to deliver instrumentally to birth in 129 cases with imminent asphyxia. One case is excluded in which the time from the decision exceeded 10 hours.

The time from the decision to deliver to birth exceeded 30 minutes in 44 of the 129 cases where both time points were noted (Figure 3). All 48 women having a spontaneous vaginal delivery had neglected pathological or noninterpretable CTG recordings for more than 45 minutes before delivery. In 6 of the 48 spontaneously delivered infants, labour was complicated by shoulder dystocia, and nine infants were born in breech presentation.

A traumatic instrumental delivery was assessed in 44 deliveries (Table 1). In 25 deliveries, trial of labour with vacuum extraction or forceps, or both, was performed before converting to caesarean section. The time from the decision to deliver to birth ranged from 13 to 88 minutes and, in 18 of these cases, it exceeded 30 minutes. For 19 of the 62 infants delivered by vacuum extraction, the time spent using the vacuum extractor exceeded 20 minutes. Four of the vacuum extractions were complicated by more than two cup detachments (Table 1).

In the 15 twin deliveries, only 1 of the twins was injured. Thirteen of these injuries occurred in the second twin. If not neglected, CTG monitoring of the second twin was based on external recordings, and there were difficulties in guiding the second twin down to the pelvic floor. The elapsed time between the births of the twins ranged from 1 minute to almost 7 hours. Four twins died—three within the first month of life.

### Neonatal outcome

The acid–base status at birth or shortly thereafter was only available in 60% (*n* = 107) of all cases. All blood samples indicated severe metabolic acidosis and profound asphyxia. The mean acid–base status was pH 6.87 and BE of −20. All 177 infants had an Apgar score <7 at 5 minutes (median 3, range 0–7). The median Apgar score was 5 at 10 minutes (range 0–7). Sixteen children had pH > 7.05 and Apgar score <7 at 5 minutes. These infants were all delivered vaginally instrumentally and were considered to suffer from postnatal asphyxia due to a traumatic mode of delivery. Forty-four percent (*n* = 78) were treated in a ventilator for more than 24 hours (median 4 days, range 1–19). Twenty-seven percent of the infants were referred to another hospital.

Information about mortality, neurological disorders co-morbidity and degree of impairment is summarised in Table 2. There were 16 neonatal deaths. Of 161 infants with severe neurological disorders, another 16 died during follow up.

**Table 2:** Mortality, neurological disorders, co-morbidity and degree of impairment

	n (%)
**Neonatal death**	16 (9)
**Neurological disorders**	
Unspecified CP syndrome	45* (25)
Dyskinetic CP	69** (39)
Spastic tetraplegia	18*** (10)
Spastic diplegia	21 (12)
Hemiplegia	8 (5)
Co-morbidity****	
Epilepsy	52 (32)
Mental restriction	54 (34)
Microcephaly	9 (6)
Severe nutritional deficiencies	38 (24)
Final degree of impairment (%)*****	
<40	10 (14)
40–79	18 (25)
80–100	45 (61)

* Nine children deceased.

** Four children deceased.

*** Three children deceased.

**** Rates are based on children alive after 28 days of age (*n* = 161).

***** Rates are based on children with information on degree of invalidity (*n* = 73).

Information about morbidity for 45 infants was based on case records with a limited follow-up period. These infants were considered to suffer from an unspecified CP syndrome with diagnosed encephalopathy during the neonatal period. Sixty nine of the 117 children with information about the type of CP had dyskinetic CP, 18 spastic tetraplegia, 21 spastic diplegia and 8 hemiplegia. Owing to the limited follow-up period, information about the final degree of impairment was available for only 73 infants. In 61% of these infants, the degree of impairment was assessed to range between 80 and 100%.

## Discussion

In this descriptive nationwide study of 177 children with severe labour-related asphyxia judged, probably to be caused by malpractice, we found insufficiencies throughout the process of treatment and care, resulting in a catastrophe for the neonate involved. At the onset of labour, fetal heart tracings were normal in all cases, indicating a nonasphyxiated fetus. As labour progressed, CTG tracings developed abnormally. This study did not intend to cover all malpractice related to delivery, but it probably mirrors acts of malpractice associated with the most severe cases of asphyxia. The results of this report must be interpreted cautiously, and some of the findings can be tested in analytical studies.

We have found that negligence in the supervision of fetal wellbeing occurred in nearly all (98%) of the pregnancies. In 71% of the pregnancies, the staff did not act timely on pathological CTGs. The most frequently observed FHR patterns associated with CP in connection with intrapartal asphyxia are those with multiple late decelerations and decreased beat-to-beat variability.^17^,^18^ These patterns are weak predictors of CP, which emphasise that we must increase our attention to all available signs of asphyxia during labour.^19^ A previous study has shown that most delivery units in Sweden have written guidelines for CTG monitoring routines and FBS.^20^ Still, despite a clear indication, FBS was not carried out in 100 of the deliveries in the present study.

Sensitivity to oxytocin increases during delivery, and there is a risk for hyperstimulation, especially late in the first stage of labour.^21^ The frequent use of oxytocin (89%), use of oxytocin without indications of labour dystocia (28%) and overdosage (39%) in women with insufficient CTG tracings in our study are alarming findings. Oxytocin causes an increase in vascular resistance in both uterine and umbilical arteries and increases the risk of a low Apgar score at 5 minutes, metabolic acidosis and transfer to the neonatal intensive care unit. A possible explanation for this could be a reduced gas exchange to the placenta caused by hyperstimulation.^22^,^23^

Despite long-standing pathological CTG tracings, the obstetrician was never called for in one-fourth of all cases. When the obstetrician was paged, the mean time from paging to birth was 73 minutes, indicating that there is also much time spent after the obstetrician has been paged. There was no way to find out whether the paged obstetricians were dealing with other emergencies, but it may indicate that there is a need to improve paging in emergency situations. When the decision to deliver was noted, only two-thirds of the infants were delivered within 30 minutes. One-fourth of them were born vaginally after neglecting signs of asphyxia, and resuscitation by skilled staff was therefore delayed.

There is a great need and a challenge to improve cooperation and to create security barriers within our labour units. The Swedish system of obstetric care, in which the midwives take all responsibility for the uncomplicated delivery, obviously makes great demands on the midwife in charge. We may have something to learn from the air force, where several mistakes must occur before an accident can happen and where the team members routinely control and check each other.^24^,^25^ Accordingly, every case of unexpected asphyxia, even those that recover without sequelae, should be investigated to enable the creation of security barriers in each labour unit. Training for unexpected and unusual situations that can happen in connection with labour, for example shoulder dystocia, might have improved the outcome for some of our children.^10^,^26^,^27^ All labour units need to identify situations that could cause misunderstandings or steal time in emergency situations. For example, we recommend checking on the effectiveness of call and alarm systems on the labour ward and how many locked doors and lifts you need to negotiate before arriving at emergency operating theatre.^28^

In 25 children, trial of labour with vacuum extraction, forceps or both was performed before converting to a caesarean delivery. This seems to have aggravated the birth trauma and the asphyxia. Five of these infants died within the first month of life and, in 13 other infants, the degree of invalidity was considered to be 95–100%. Nineteen of 62 infants delivered by vacuum extraction were delivered after more than 20 minutes and four had more than two cup detachments. When immediate delivery is requested because of imminent asphyxia, both obstetricians and midwives must be confident in choosing the fastest and less traumatising way to deliver, taking both maternal and infant health into account.^11^,^29^

The International Cerebral Palsy Task Force uses the following criteria to define CP caused by asphyxia in relation to delivery: (i) metabolic acidosis in umbilical arterial cord or very early infant blood samples, pH < 7 and base deficit >12 mmol/l; (ii) a sentinel hypoxic event before or during labour, (iii) early onset of severe or moderate encephalopathy in infants born at 34 weeks or later; (iv) CP of the spastic quadriplegic or dyskinetic type and exclusion of other identifiable aetiologies, such as trauma, coagulation disorders, infectious conditions or genetic disorders.^16^ In our definition of asphyxia in connection with delivery, we were hampered by inadequacies in the available documentation, including CTG recordings and fetal, umbilical cord and infant blood sampling. It is likely that most of our cases involved a sentinel hypoxic event as an intrapartum hypoxic event can either be silent or evident.^18^ When available (60%), the acid–base status at birth indicated profound metabolic acidosis and asphyxia, and all infants had an Apgar score at 5 minutes of <7 (median 3). A low Apgar score at 5 minutes correlates well with asphyxia in the absence of malformations.^30^

Some intrapartum factors may be single cause of encephalopathy for which reason the definition labour-related brain damage should be used with caution.^14^ However, a vulnerable cerebral status of a fetus may be aggravated by a hypoxic event during delivery. In the study group, there were 16 asphyxia-related neonatal deaths, 45 infants with an unspecified CP syndrome (due to a limited follow-up period) and 116 with a CP diagnosis. Among the children with an unspecified CP syndrome, 20% died before a final diagnosis could be achieved. Among the children with diagnosed CP, 74% had either dyskinetic CP or spastic tetraplegia, the only subtypes of CP that are associated with intrapartum hypoxic events.^18^ Although some infants in the study group may have had encephalopathy prior to labour, we find it probable that delivery-related asphyxia may have aggravated the encephalopathy in these cases.

## Conclusion

On scrutinising our cases of severe asphyxia in connection with delivery, we conclude that fetal surveillance and attention to signs of asphyxia must be improved. Every case of unexpected asphyxia, even those that recover without sequelae, can be used as a learning experience for creating barriers of security in each labour unit.

## Contribution to authorship

The study was planned by all authors. S.B. collected all data, made the main part of analyses and drafted the manuscript. S.C., C.G. and H.P. assisted in analyses, interpreting of results and revisions of the manuscript. The final version of the manuscript was approved by all authors.

**Journal club**

Expectation and experiences of childbirth in primiparae with caesarean section.

This nationwide descriptive study from Sweden examines in detail the events surrounding labour in 472 women asking for financial compensation because of suspected medical malpractice in connection with childbirth during 1990–2005. They concluded that the most important causes of malpractice were neglecting to supervise fetal wellbeing, neglecting signs of fetal asphyxia, inappropriate use of oxytocin and choosing a nonoptimal mode of delivery.

**Discussion points**

1. Background: Are these findings surprising? Are there other studies that have shown similar factors are responsible for adverse outcomes?
2. Technical: Do you think that the methodology of this study is appropriate and allows the conclusions that they have drawn? What is the value of the ‘admission CTG’ and what is the evidence for its beneficial use in low-risk women in labour.
3. Clinical practice: Do you think that the study has findings that should influence CTG and labour management training schemes for obstetricians and midwives? In this respect, which of the findings of this study do you think are most important.
4. Future research: How would you go about investigating the contribution of psychological and institutional factors in: (a) supervision of fetal wellbeing and (b) neglect of the signs of fetal asphyxia.

*Correspondence:*Dr M Marsh, Denmark Hill, London SE5 9RS, UK. Email michael.s.marsh@kcl.ac.uk
